# Targeting Wnt Pathways with Small Molecules as New Approach in Cardiovascular Disease

**DOI:** 10.2174/011573403X333038241023153349

**Published:** 2024-10-30

**Authors:** Seyed Mehdi Mousavi, Fatemeh Jalali-Zefrei, Mohammad Shourmij, Shiva Tabaghi, Amirhesam Davari, Saeed Bahador Khalili, Soghra Farzipour, Arsalan Salari

**Affiliations:** 1 Cardiovascular Diseases Research Center, Department of Cardiology, School of Medicine, Heshmat Hospital, Guilan University of Medical Sciences, Rasht, Iran;; 2 Department of radiology, Faculty of Medicine, Guilan University of Medical Science, Rasht, Iran;; 3 Razi Herbal Medicines Research Center, Lorestan University of Medical Sciences, Khorramabad, Iran;; 4 Cardiovascular Research Center, Shahid Beheshti University of Medical Sciences, Tehran, Iran;; 5 Department of Electronic Engineering, Universitat Rovira i Virgili, Tarragona, 43007, Spain

**Keywords:** Wnt pathway, cardiovascular disease, Wnt inhibitors, β-catenin, canonical pathway, non-canonical pathway

## Abstract

The increasing incidences of morbidity and mortality associated with cardiovascular diseases represent significant difficulties for clinical treatment and have a major impact on patient health. Wnt signaling pathways are highly conserved and are well known for their regulatory roles in embryonic development, tissue regeneration, and adult tissue homeostasis. Wnt signaling is classified into two distinct pathways: canonical Wnt/β-catenin signaling and non-canonical pathways, including planar cell polarity and Wnt/Ca^2+^ pathways. A growing body of experimental evidence suggests the involvement of both canonical and non-canonical Wnt signaling pathways in the development of cardiovascular diseases, including myocardial hypertrophy, arrhythmias, diabetic cardiomyopathy, arrhythmogenic cardiomyopathy, and myocardial infarction. Thus, to enhance patient quality of life, diagnosing and treating cardiac illnesses may require a thorough understanding of the molecular functions played by the Wnt pathway in these disorders. Many small-molecule inhibitors specifically target various components within the Wnt signaling pathways, such as Frizzled, Disheveled, Porcupine, and Tankyrase. This study aims to present an overview of the latest findings regarding the functions of Wnt signaling in human cardiac disorders and possible inhibitors of Wnt, which could lead to novel approaches for treating cardiac ailments.

## 1 INTRODUCTION

Wnt signaling pathways are highly conserved due to their well-established regulatory roles in adult tissue homeostasis, tissue regeneration, and embryonic development. These pathways are categorized into canonical and non-canonical Wnt signaling pathways. The canonical Wnt/β-catenin signaling pathway is reliant on β-catenin, while the Wnt/Ca^2^+ and planar cell polarity (Wnt/PCP) pathways constitute the β-catenin-independent non-canonical Wnt pathways. The cardiovascular system is among the first functional systems to form during embryogenesis, and its development is significantly influenced by WNT signaling mediated by both β-catenin and non-β-catenin pathways. The multi-step process that leads to the formation of the heart begins with the specification of cardiac progenitor cells and the generation of the heart tube. Subsequent steps include cardiac looping, chamber formation, septation, and maturation. Wnt proteins regulate critical processes such as proliferation, migration, and differentiation during both embryonic and adult development, making them essential for cardiovascular system formation [1]. Numerous studies have demonstrated that the protein expression levels of canonical and non-canonical signaling pathways significantly increase in damaged hearts and various cardiovascular diseases. Therefore, inhibiting these pathways presents a promising strategy for reducing the incidence of cardiovascular diseases. The purpose of this study is to provide an overview of the roles played by both the canonical and non-canonical Wnt pathways in cardiac disorders, as well as to discuss recent inhibitors of these pathways that may offer novel therapeutic approaches for treating cardiac ailments.

## 2 WNT/PATHWAY

Wnt is a portmanteau derived from “int-1,” a gene found in mammals, and “wingless,” a crucial segmentation gene in Drosophila. This protein family comprises nineteen glycoproteins that bind to Frizzled (Fzd) family receptors. In humans, there are ten distinct variations of Fzd, and the associated proteins exhibit seven transmembrane domains, classifying them as members of the G protein-coupled receptor family. Upon binding of Wnt to Fzd, three distinct signaling pathways are activated in the cytoplasm: the Wnt-calcium (Wnt/Ca^2+^) pathway, the non-canonical planar cell polarity (Wnt/PCP) pathway, and the canonical (β-catenin-dependent) Wnt pathway [2].

### 2.1 Canonical Wnt/β Signaling

The canonical Wnt pathway is vital for many bodily functions, including organogenesis, bone metabolism, and cell fate. When inactive, a protein complex comprising Axin, adenomatous polyposis coli (APC), and glycogen synthase kinase-3 (GSK3) regulates β-catenin phosphorylation [3]. This leads to β-catenin being marked with ubiquitin and degraded by proteasomes. When Wnt signaling is activated, Wnt binds to low-density lipoprotein receptor-related protein 1 (LRP1) and Fzd receptors, triggering Dvl. Dvl then inhibits GSK3, causing the breakdown of the Axin-APC-GSK3 complex, resulting in β-catenin accumulation in the cytoplasm. Once in the nucleus, β-catenin interacts with proteins such as the T-cell factor/lymphoid enhancer-binding factor 1 (LEF1) family, allowing it to activate or repress target Wnt genes. The presence or absence of specific receptors influences cell fate, leading to different complexes forming through interactions with Wnt molecules [4, 5].

### 2.2 Non-Canonical Pathway

Wnt/PCP and Wnt/Ca^2+^ are the two main noncanonical, β-catenin-independent Wnt signaling pathways. Other noncanonical pathways include Wnt-mTOR, Ror2, RAP1, PKA, JNK, GSK3MT, and RYK, which exhibit some overlap. Wnt4 and Wnt5a are thought to participate in these noncanonical cascades, while Wnt1, Wnt3a, Wnt8, and Wnt8b are involved in the Wnt/β-catenin signaling cascade [6]. In the Wnt/PCP pathway, the GTPases Rac and Rho regulate cytoskeletal changes, leading to lateral asymmetry. In the Wnt/Ca^2+^ pathway, Wnt binds to Fzd, enhancing its interaction with the Dvl/Axin/GSK3 complex. GSK3 facilitates the phosphorylation of Wnt-Fzd coreceptors Ror1 and Ror2, activating phospholipase C, which cleaves phosphatidylinositol 4,5-bisphosphate into 1,2-diacylglycerol (DAG) and inositol 1,4,5-triphosphate (IP3). Elevated Ca^2+^ levels result from IP3-activated calcium channels on the endoplasmic reticulum (ER), triggering the activation of calmodulin-dependent protein kinase II [7]. Moreover, DAG activates protein kinase C *via* released Ca^2+^ from the ER. Numerous nuclear transcription factors are activated by these kinases and released Ca^2+^ may also activate the protein phosphatase calcineurin, which can activate the cytoplasmic nuclear factor associated with T cells. Ultimately, the transcription of target Wnt genes is influenced by nuclear factors translocating into the nucleus [8].

## 3 WNT/β CATENIN SIGNALING IN CARDIOVASCULAR DISEASE

In human infarcted hearts, the Wnt/β-catenin pathway is activated, while it remains inactive in healthy hearts. Reports indicate that this pathway is not activated during the first 24 hours post-myocardial infarction, gradually becoming active, peaking at 7 days, and disappearing after 3 weeks [9]. Changes in the body's defense mechanisms against oxygen free radicals during myocardial infarction and cardiac reperfusion can lead to oxidative stress and damage [10]. An *in vitro* model of oxidation-damaged cardiomyocytes demonstrated activation of the Wnt/β-catenin pathway. Reactive oxygen species (ROS) can activate the PI3K/AKT pathway by suppressing the phosphatase and tensin homolog (PTEN). Phosphorylated AKT then phosphorylates GSK-3β, which activates the Wnt/β-catenin pathway. Additionally, oxidative stress can activate this pathway by disrupting the binding of nucleoredoxin, a thioredoxin family member, to the Dvl PDZ domain, leading to increased stability of cytoplasmic β-catenin and enhanced downstream Wnt signaling [11]. Liu *et al*. found that oxidative stress-induced DNA damage and p53-mediated apoptosis were exacerbated by activation of the nuclear β-catenin/c-Myc axis. In contrast, inhibiting the Wnt/β-catenin pathway reduced oxidative stress-induced DNA damage and apoptosis. Furthermore, proteins associated with apoptosis, such as Bax, cytochrome c, and caspase-3, were more prevalent in cardiomyocytes transfected with β-catenin plasmids. In β-catenin knockout mice with myocardial infarction, levels of active caspase-3 and Bax were significantly lower, while anti-apoptotic Bcl-2 levels were higher [12]. These results suggest that β-catenin may exacerbate oxidative stress-induced damage.

Normal cardiac macrophages do not display the Wnt/β-catenin pathway; however, after myocardial infarction, Ly6C+ proinflammatory macrophages show elevated levels of dissociated β-catenin in their cytoplasm [13]. Activated β-catenin enhances the expression of pro-inflammatory cytokines, such as IL-1β, IL-6, TNF-α, and IL-23 p19, potentially promoting macrophage-mediated inflammation. Additionally, β-catenin interacts with NF-κB, a crucial transcription factor in the inflammatory response, which stimulates the production of inflammatory enzymes and proteins. In cardiomyocytes, β-catenin overexpression is associated with increased expression of inflammatory markers, including TNF-α, p-NF-κB, and IL-8, along with the accumulation of NF-κB in the nucleus, suggesting that β-catenin activates NF-κB to facilitate inflammation. Although the relationship between β-catenin and inflammation is complex, β-catenin may serve as a potential regulatory target for inflammatory responses following myocardial infarction [14].

A significant contributor to myocardial fibrosis is β-catenin. Liu *et al*. found that fibroblasts exhibit upregulation of various fibrosis markers upon β-catenin overexpression. Additionally, Bing Zou *et al*. demonstrated that activation of the Wnt/β-catenin pathway is linked to improved cardiac function in hypoxic cell models and myocardial infarction (MI) mice [15]. Elevated β-catenin levels may trigger epithelial-mesenchymal transition (EMT), which plays a critical role in the generation of muscle fiber cells and angiogenesis, as well as tissue healing. In animal models, two weeks post-acute ischemic heart injury, Wnt1, and β-catenin expression increased in epicardial cells and fibroblasts. Epicardial cells undergoing EMT exhibited heightened expressions of profibrotic genes (ET-1, Col1, and Col3), promoting fibroblast development. Disruption of the Wnt/β-catenin pathway in these cells can result in EMT deficiency, reduced fibroblast proliferation, epicardial dilatation, and impaired cardiac function [16].

β-catenin overexpression also triggers fibroblast DNA replication, increases fibroblast numbers, and enhances fibrosis in myofibroblasts *via* transforming growth factor-β1 (TGF-β), which mediates fibroblast-to-myofibroblast differentiation in response to mechanical damage and environmental stimuli. The Wnt/β-catenin pathway boosts interleukin-11 (IL-11) production, further facilitating TGF-β-driven fibroblast transition [17]. Multiple studies suggest that early inhibition of the Wnt/β-catenin pathway during myocardial infarction can reduce myocardial fibrosis, limit infarct size, and improve myocardial remodeling and cardiac function. Therefore, targeting β-catenin may provide a strategy to alleviate myocardial fibrosis and cardiac failure after infarction [18, 19]. In rats, Wnt2 and Wnt4 levels significantly increased on the third-day post-MI, correlating with rises in TGF-β1, Col1, and Col3. Overexpression of Wnt2/Wnt4 induced fibrosis through the β-catenin/NF-kB/p65 pathway, requiring Fzd4/Fzd2 and LRP6. Inhibition of β-catenin with ICG-001 blocked this pathway and mitigated fibrosis effects.

Arrhythmias arise from structural or electrical abnormalities, often due to acquired factors like heart failure and cardiovascular disease (CVD). Disruptions in cardiac gap junctions, particularly involving connexin43 (Cx43), can lead to conduction anomalies. The Wnt/β-catenin pathway regulates Cx43 expression and interacts with it to influence arrhythmia risk. Disruption of the β-catenin/cadherin complex results in gap instability, potentially causing arrhythmias [20].

The Nav1.5 protein, crucial for Na^+^ channel function, is also influenced by the Wnt/β-catenin pathway. Enhancing the Wnt/β-catenin/TCF4 pathway can increase arrhythmia susceptibility by suppressing Nav1.5 expression, leading to arrhythmias during the acute phase of myocardial infarction (MI), while chronic remodeling occurs in later phases. Studies indicate a reduction in arrhythmias in β-catenin-deficient animals post-MI, suggesting changes in ion channel gene expression [21]. Diabetic cardiomyopathy (DCM) is characterized by heart failure, hypertrophy, and myocardial dilatation, with mechanisms involving oxidative stress, inflammation, and apoptosis. Elevated levels of Wnt2, β-catenin, and c-Myc in DCM models could mitigate diabetic heart failure, highlighting β-catenin as a potential therapeutic target. Inhibition of the β-catenin/TCF4/GSK-3β/mTOR pathway may enhance autophagy and reduce DCM severity [22]. Myocardial hypertrophy (MH) is an adaptive response to external stimuli, marked by cell enlargement and expression of fetal genes such as brain natriuretic peptide (BNP) and atrial natriuretic peptide (ANP). β-Catenin overexpression is linked to MH, while its deletion or inhibition can mitigate it. The Wnt/β-catenin pathway plays a significant role in MH, with β-catenin overexpression leading to increased cell volume and activation of pathological hypertrophy markers (p38, JNK1/2, ERK1/2, ERK5) [23]. In a mouse model of Ang II-induced MH, activation of the Wnt/β-catenin pathway increased β-catenin and c-Myc levels, with observations of nuclear β-catenin accumulation and decreased phosphorylated β-catenin as well as elevations in inactive GSK3β (phosphorylation at ser9).

Inhibiting the Wnt/β-catenin pathway with ICG-001 effectively reduces cardiac hypertrophy induced by Ang II [24]. According to research by Wang *et al*., acute activation of this pathway may enhance prosurvival gene expression and induce metabolic reprogramming, offering cardioprotective benefits during myocardial infarction. However, persistent activation can lead to fibrosis and hypertrophy in the adult heart, highlighting the importance of spatiotemporal regulation of Wnt/β-catenin signaling for proper cardiac development [25, 26]. Research indicates that Wnt/β-catenin signaling is essential for early vertebrate heart development, evidenced by studies showing ectopic heart formation in embryos with conditional β-catenin inactivation. While this pathway initially drives differentiation, its role decreases later in development, as seen in studies involving zebrafish, mice, and human embryonic stem cells [27]. Additionally, β-catenin has been shown to promote cardiomyocyte proliferation in zebrafish and mice [28]. β-Catenin influences cardiac growth and function by participating in cardiomyocyte metabolism [29]. It may affect the utilization of glucose and fatty acids, impacting prenatal cardiometabolic maturation. In perinatal cardiomyocytes, β-catenin ablation leads to mitochondrial insufficiency, and heterozygous β-catenin knockout mice exhibit an inability to increase mitochondrial numbers in response to exercise training. Furthermore, research by Stubenvoll *et al*. demonstrated that ablation of emerin-an inhibitor of β-catenin nuclear import-exacerbates cardiac remodeling after pressure overload, leading to heart failure [30, 31].

Numerous studies have revealed that both canonical and non-canonical Wnt signaling pathway protein expressions increase significantly in damaged hearts and cardiovascular diseases. Therefore, inhibiting these pathways presents a promising strategy for mitigating cardiovascular diseases. This review aims to explore the role of Wnt signaling pathways in various cardiovascular conditions and introduce small molecule inhibitors of canonical and non-canonical Wnt pathways that may play a critical role in regulating cardiovascular health. Table **1** highlights key members of canonical and non-canonical Wnt signaling associated with cardiovascular disease.

## 4 NON-CANONICAL WNT SIGNALING IN CARDIOVASCULAR

Numerous pathogenic pathways, including arrhythmogenesis, dysregulated cardiac biomechanics, structural remodeling, aberrant energetics, and oxidative stress, are responsible for the development of cardiac illnesses, including heart failure (HF) and arrhythmias [32, 33]. There is observational evidence linking heart illnesses to non-canonical WNT signaling. For instance, circulating levels of WNT5A were higher in heart failure patients than in non-HF patients, and they were linked to hemodynamic indicators of HF as filling pressure and ejection fraction.

In a group of patients with dilated cardiomyopathy, higher plasma WNT5A levels were associated with higher right ventricular filling pressures and a lower right ventricular ejection fraction. Furthermore, compared to the left ventricle, the right ventricle had greater WNT5A expression. Arrhythmogenic right ventricular cardiomyopathy (Arc) has been associated with classical β-catenin WNT signaling as well as non-canonical RHO-mediated WNT signaling, according to *in silico* models. It has been shown that elevated PPARγ expression and ARVC pathogenesis are associated with the inactivation of these pathways [34]. On the other hand, not enough research has been done on the molecular significance of non-canonical WNT signaling in human arrhythmogenesis. In a research including patients with rheumatic valve disease undergoing valve surgery, atrial fibrillation was associated with increased expression of the transcription factor SNAIL1 and other WNT ligands, including the non-canonical WNT ligands WNT5A and WNT11, in the right atrium [35]. In research including patients with rheumatic valve disease undergoing valve surgery, atrial fibrillation was associated with increased expression of the transcription factor SNAIL1 and other WNT ligands, including the non-canonical WNT ligands WNT5A and WNT11, in the right atrium [36, 37].

These results thus suggest that non-canonical WNT signaling plays a causal role in arrhythmogenesis by promoting the growth of re-entrant circuits. It's possible that non-canonical WNT signaling causes heart fibrosis. WNT5A, for instance, it has been demonstrated to activate ERK1/ERK2 signaling and produce IL-6, tissue inhibitor of metalloproteinase 1 (TIMP1), and human primary cardiac fibroblasts *in vitro* [38]. Some research implies that WNT5A may encourage fibrosis and inflammation *in vivo*. Additionally, WNT5A lowers the amounts of glycogen synthase kinase 3β (GSK3β) in human cardiac fibroblasts *in vitro*, which partially increased fibrosis through TGFβ transactivation, however traditional WNT signaling appeared to be the primary mediator of this action [39]. The NFAT–calcineurin pathway is activated in human ventricular cardiomyocytes through noncanonical [40], Ca^2+^-dependent WNT signaling, which has been linked in experimental models to cardiac fibrosis. It has been demonstrated that non-canonical WNT signaling promotes cardiomyocyte hypertrophy [41]. More precisely, as determined by microscopy and cardiomyocyte surface area [42]; WNT5A increased PCP signaling mediated by Dapper 1 and resulted in downstream activation of JNK to induce hypertrophy in human cardiomyocytes *in vitro*. Interestingly, myeloid-specific Wnt5a knockdown and neutrophil depletion both decreased cardiac hypertrophy and neutrophil infiltration in a mouse model of left ventricular hypertrophy brought on by aortic constriction [43]. This discovery implies that wnt5A may indirectly control ventricular hypertrophy through neutrophils and the heart’s local inflammatory response.

Arrhythmogenesis and contractile dysfunction are just two examples of cardiac disease phenotypes that are associated with fatty infiltration of the myocardium. There is no information on the relationship between non-canonical WNT signaling and fatty remodeling of the myocardium, even though inhibition of conventional WNT signaling has been connected to fatty infiltration of the myocardium, for instance, in animal models of ARVC, Canonical WNT3A signaling was able to save a mouse model of non-alcoholic fatty liver disease, where non-canonical WNT signaling has been linked to fatty infiltration in other organs [44-46]. Research indicates that WNT signaling, mostly through canonical signaling, is responsible for metabolic pathway reprogramming in a range of cell types. Through PKC and control of intracellular Ca^2+^ dynamics, non-canonical WNT signaling inhibits mitochondrial aggregation in the HEK93 cell line1, shields mitochondria against fission–fusion changes, and stops mitochondrial loss in neurons [47, 48]. Through processes involving VEGF and insulin-like growth factor 1, overexpression of the non-canonical WNT ligand WNT11 maintains mitochondrial membrane potential and shields cardiomyocytes from hypoxia *in vitro*. Additionally, non-canonical WNT signaling quickens the liver's glucose oxidative metabolism, which triggers steatosis by de novo lipogenesis. The function of non-canonical WNT signaling in myocardial energetics has not been thoroughly investigated, although these results suggest connections to cardiac metabolism [49]. In cardiac disorders such as ischemia-reperfusion damage following myocardial infarction and heart failure and arrhythmias through redox signaling, oxidative stress plays a crucial pathophysiological role. Ischemia-reperfusion injury, inflammation-mediated activation of NADPH oxidases, and imbalanced mitochondrial energy, all lead to oxidative stress. In turn, myocardial ROS can cause hypertrophy, apoptosis, autophagy, dysregulation of metabolic enzymes, and decreased contractility, all of which have a significant negative impact on myocardial disease [50, 51]. The hallmark of ischemia-reperfusion injury is abrupt availability of oxygen in a shocked myocardial, where pro-oxidant and antioxidant enzymes are out of balance, resulting in the production of free radicals. Through a variety of intracellular mechanisms, such as proteolysis, caspase activation, and mitochondrial regulation, the excess free radicals cause cardiomyocyte apoptosis [52]. Hypoxia-reperfusion damage is lessened and mesenchymal stem cell proliferation and differentiation into cardiomyocytes are stimulated when AKT1 and the non-canonical ligand WNT11 are co-expressed. In contrast, a study conducted on mice revealed that myocardial ischemia-reperfusion damage is lessened when non-canonical WNT signaling is blocked. Numerous redox-sensitive intracellular transcription pathways have linked NADPH oxidases to atrial fibrillation and heart failure. RAC1 connects non-canonical WNT signaling to NADPH oxidases, as demonstrated *in vitro* in VSMCs15. It remains to be shown if these findings apply to the human heart [53, 54].

## 5 WNT/β-CATENIN AND PATHWAY INHIBITORS FOR CARDIAC DISEASES

Given its potential role in various diseases-including cancer, neurodegenerative disorders, and cardiovascular diseases-numerous compounds have been developed to target the Wnt signaling pathway. While many of these compounds focus on the WNT/β-catenin branch, some act on the receptor complex or influence WNT synthesis and secretion, which occur further upstream. In the following section, we will introduce several strategies employed to target the Wnt signaling pathway, highlighting a selection of Wnt inhibitors in Table **2** that detail their effects and targets specifically in cardiovascular diseases.

### 5.1 WNT Synthesis-Secretion

Numerous PORCN inhibitors have been identified that impact WNT secretion and mediate the inhibition of WNT signaling activation. The synthesis and secretion of WNT ligands constitute the initial stage of the WNT signaling cascade. PORCN plays a crucial role in the palmitoylation of WNT proteins, a modification essential for their stability and proper release. After this modification, WNTLESS (WLS) facilitates the trafficking and export of WNTs from the producing cells into the extracellular space, ensuring effective signaling. In addition to these proteins, several small molecules have been developed that target WNTLESS, such as *LGK974* and *IWP-2*. These compounds function as WLS inhibitors and can disrupt the trafficking process of WNT proteins, thereby providing potential therapeutic avenues for manipulating WNT signaling in various diseases. Table **2** illustrates some of the PORCN inhibitor compounds, which have proven effective against various types of cancer in both *in vitro* and *in vivo* studies. However, there is a lack of studies investigating their effectiveness in cardiovascular diseases. Recently, WNT-C59 has emerged as a promising drug for patients with cardiac hypertrophy. In mice undergoing transverse aortic constriction (TAC) surgery, the addition of WNT-C59 resulted in significantly improved cardiac function and a higher survival rate. Histologically, WNT-C59 reduced TAC-induced increases in heart mass, cardiomyocyte cross-sectional area, cardiac fibrosis, cardiomyocyte apoptosis, and the expression of hypertrophic biomarkers such as β-MHC, ANP, and BNP. Additionally, WNT-C59 mitigated oxidative damage associated with TAC. It also inhibited *in vitro* cardiomyocyte hypertrophy induced by Angiotensin II, as evidenced by smaller cell sizes and reduced production of β-MHC, BNP, and ANP [67].

Jiang *et al*. demonstrated the anti-hypertrophic effects of the novel small-molecule porcupine inhibitor CGX1321, which is currently undergoing clinical trials in humans as an anti-cancer therapy. In mouse models subjected to transverse aortic constriction (TAC), CGX1321 significantly improved both cardiac function and animal survival. Additionally, CGX1321 markedly reduced cardiomyocyte hypertrophy, apoptosis, and fibrosis induced by TAC injury. The compound exhibited a potent inhibitory effect on the canonical WNT signaling pathway, as evidenced by the inhibition of TAC-induced nuclear translocation of β-catenin. Furthermore, it led to a decrease in the expression of Frizzled-2, cyclin-D1, and c-myc, which are crucial mediators in the WNT signaling cascade [68].

Wu *et al*. recently demonstrated that CGX1321 therapy markedly reduced cardiac hypertrophy and fibrosis, resulting in improved exercise performance and enhanced cardiac diastolic function in animal models of heart failure with preserved ejection fraction (HFpEF). The treatment effectively suppressed both canonical and non-canonical WNT signaling pathways, as evidenced by decreased phosphorylation of c-Jun and reduced nuclear translocation of β-catenin and NFATc3. Additionally, CGX1321 therapy inhibited the production of WNT ligands, further contributing to its therapeutic effects [69].

In their *in vivo* investigation, Meyer *et al*. demonstrated that animals treated with LGK-974 exhibited enhanced cardiac function and a reduced inflammatory response. Specifically, LGK-974 suppressed Wnt signaling activation in monocytes and macrophages, leading to a decrease in their pro-inflammatory phenotype in an *in vitro* model of hypoxic cardiomyopathy and monocyte/macrophage interaction. The study elucidated the impact of Wnt signaling on the inflammatory cascade following myocardial infarction (MI). As a Wnt secretion inhibitor, LGK-974 appears to be a promising candidate for future immunomodulatory strategies aimed at improving cardiac remodeling after MI [70].

Recently, Nayakanti *et al*. demonstrated that LGK-974 attenuated fibrosis and cardiac hypertrophy, resulting in improved right ventricular (RV) function in both pulmonary artery banding and monocrotaline-induced RV overload models in mice. Furthermore, Bastakoty *et al.* [71] showed that pharmacologic suppression of the WNT pathway through intravenous injection of GNF-6231 after myocardial infarction (MI) significantly decreased the deterioration of myocardial function, prevented unfavorable cardiac remodeling, and reduced myocardial size in C57BL/6 mice. These findings highlight the potential of targeting WNT signaling pathways in developing therapeutic strategies for improving cardiac performance following various types of cardiac stress [71].

### 5.2 Extracellular Pharmacological Targeting of Wnt Signaling: WNT-FZD Interaction

The binding of Wnt to the Frizzled (FZD) receptor initiates the signaling transmission. Various compounds have been tested for their effect on this binding. Blocking FZD receptors with antibodies is an appealing therapeutic approach due to the high affinity and specificity with which antibodies typically bind their targets. Several FZD isoforms have been targeted by antibodies developed by different organizations. Pharmacological interventions may influence the signaling cascade through interaction with the LRP5/6 coreceptor, as phosphorylation of LRP6 is a critical component of WNT-mediated activation. Recently, it has been demonstrated that vantictumab, also known as antibody OMP-18R5, holds promise for inhibiting various FZD receptor targets. OMP-18R5 has shown efficacy when used in conjunction with first-line chemotherapy and against a variety of human malignancies, including teratocarcinoma, lung, pancreatic, and breast cancers. *In vitro* and *in vivo* anticancer activities have been reported for most of these antibodies, underscoring their potential as therapeutic agents in oncology [92, 93].

Sclerostin, a protein that binds to LRP4, LRP5, and LRP6, has recently been shown to inhibit Wnt signaling. This inhibition reduces the effects of angiotensin II (AngII) on atherosclerosis and aortic aneurysm. The mechanism involves a decrease in macrophage infiltration and a reduction in the expression of genes responsive to β-catenin. These findings support the idea that targeting the Wnt pathway could limit or halt the development of atherosclerotic lesions, highlighting its potential as a therapeutic strategy [94].

IGFBP-4/H95P has been shown to inhibit β-catenin activation both *in vitro* and *in vivo*, preventing the occurrence of DNA damage. In contrast, high serum levels of Dkk1 are associated with premature myocardial infarction (MI) in humans. While β-catenin exacerbates ischemic injury, the integral basal signaling of LRP5/6 is crucial for protecting against myocardial ischemia damage. Interestingly, although Dkk1 inhibits the overexpression of nuclear β-catenin and downregulates LRP5/6, its overall effect appears to enhance myocardial ischemia injury through further downregulation of LRP5/6. In contrast, IGFBP-4/H95P does not affect basal LRP5/6 signaling; rather, it prevents the overexpression of β-catenin during ischemic conditions. Consequently, IGFBP-4/H95P offers protective effects against ischemic damage in the heart [95, 96]. Previous research by Foulquier *et al*. showed the significant effects of UM206, a 13-amino acid-long peptide fragment of WNT3A/WNT5A, on FZD-receptor blockage, which results in the suppression of the WNT signaling pathway [97]. Two cysteine (Cys) residues are present in UM206, and their replacement with another amino acid has been shown to adversely affect the molecule's activity. This suggests a potential covalent binding between the two Cys in UM206 and the Cys-rich region of the frizzled (FZD) receptors. Additionally, UM206 has demonstrated inhibitory effects on FZD1 and FZD2 receptors in the presence of WNT3A, but not on FZD3 or FZD4, indicating its selectivity.Researchers decided to investigate the effects of UM206 after myocardial infarction (MI) in mice due to its significant impact on the WNT signaling pathway. Thirty-five days post-MI, UM206 therapy showed beneficial effects on cardiac remodeling and prevented the development of heart failure (HF). This effect was associated with an increase in the number of myofibroblasts in the infarct area and enhanced scar healing. Notably, while the myofibroblast count was lower in this study, decreased left ventricular dilation and improved infarct repair were observed in a pig model of MI where UM206 was administered for five weeks after the event. Moreover, soluble frizzled-related protein 1 (SFRP1), which features a cysteine-rich domain similar to that of frizzled receptors, selectively targets circulating Wnt ligands. SFRP1 inhibits the transduction of the WNT/β-catenin pathway into cells, thereby negatively regulating this crucial signaling pathway [98]. According to prior research, overexpression of soluble frizzled-related protein 1 (SFRP1) can effectively reduce the activity of the Wnt/β-catenin signaling pathway and decrease the mortality rate associated with chronic heart failure in older mice. SFRP1 inhibits this pathway, providing significant protection to aging hearts from acute myocardial infarction (AMI) injury.As a small molecule gene therapy, SFRP1 has the potential to enhance heart function, reduce myocardial fibrosis, block cardiomyocyte apoptosis, and mitigate AMI injury in aged animals. These findings suggest that SFRP1 could be a promising therapeutic agent for improving cardiac outcomes in older patients suffering from heart-related conditions [99].

### 5.3 Intracellular Compartment

#### 5.3.1 Interventions Targeting Dishevelled

Dishevelled (Dvl) proteins are crucial regulators of the Wnt signaling cascade, interacting with the Wnt receptor Frizzled through their PDZ domains. Upon binding of WNT proteins to Frizzled receptors, the scaffold protein Dishevelled (Dvl) becomes activated. This activation leads to the inhibition of the serine/threonine kinase GSK3-β *via* the canonical Wnt signaling pathway. The inhibition of GSK3-β subsequently triggers β-catenin-mediated transcription of target genes. One potential strategy for cancer treatment involves blocking the Dvl PDZ–Frizzled interaction, which has spurred the development of small-molecule inhibitors such as Ky-0232 and the anti-inflammatory drug Sulindac. However, emerging research indicates that cardiac overexpression of Dishevelled 1 (Dvl-1) is associated with the development of cardiomyopathy. Although various small molecules have been designed to target Dvl for cancer inhibition as summarized in Table **2**, to date, there has been no research investigating their applicability in cardiovascular diseases. This presents an important avenue for future research, as exploring the effects of these inhibitors in cardiac contexts could reveal new insights into both cancer and heart disease treatment [100]. KY-02327 is recognized as a potent enzyme inhibitor within the context of cancer treatment. In addition, various sulfonamide derivatives have been specifically designed to inhibit the Dvl-PDZ pathway, demonstrating stronger effects than KY-02327 *in vitro*. Table **2** illustrates the chemical structures of KY-02327 alongside several sulfonamide derivatives that target Dishevelled proteins, highlighting their potential applicability in cardiovascular disease (CVD) therapy.

#### 5.3.2 Interventions Targeting Axin

The β-catenin destruction complex is primarily composed of adenomatous polyposis coli (APC) and Axin, which work together to promote the degradation of β-catenin. Tankyrases, members of the PARP family of poly-ADP-ribosylation enzymes, play a crucial role in regulating the levels of Axin. Inhibition of tankyrase activity weakens Wnt signaling while enhancing the stability of Axin. Specifically, tankyrases 1 and 2 (TNKS1) facilitate the degradation of β-catenin by poly (ADP-ribosyl) ation (PARsylation) of Axin. When tankyrase inhibitors are utilized, Axin stability increases, leading to enhanced β-catenin degradation and reduced Wnt/β-catenin signaling activity. Given their pivotal role, TNKS inhibitors have been investigated for various disorders, including fibrotic diseases, infections caused by Epstein-Barr and Herpes simplex viruses, as well as cancers of the colon, lung, and prostate, and conditions like cherubism and systemic sclerosis. Recent studies also suggest that using silico methods to induce heart regeneration following injury may present a promising therapeutic avenue [101]. Wang *et al*. studied the expression and activation of tankyrases (TNKSs) in the myocardium of myocardial infarction (MI) rats and ischemic heart failure (IHF) patients. Their research explored the cardioprotective potential of TNKS inhibition using a zebrafish model of heart failure induced by isoproterenol. The findings revealed elevated levels of DICER and TNKS2 in IHF patients, alongside an overexpression of miR-34a-5p and miR-21-5p in the non-infarcted myocardium. In the early stages following MI in a rat model, increased levels of TNKS2 and DICER were observed in both the border and infarct areas. Moreover, from four weeks post-MI, the researchers noted destabilization of Axin in the infarct area and persistently elevated TNKS1 levels in the border and infarct regions, which stimulated Wnt/β-catenin signaling [102]. Table **2** demonstrates chemical structure of XAV939 targeting axin for cardiovascular disease.

#### 5.3.3 Interventions Activating GSK3b

Following the activity of one or more priming kinases, glycogen synthase kinase 3 (GSK-3), a widely expressed serine/threonine protein kinase, phosphorylates multiple sites in the N-terminal regulatory domains of NFAT proteins. [103]. Glycogen synthase kinase 3 (GSK-3) is highly active in unstimulated cells but becomes inactivated in response to mitogenic stimulation, which distinguishes it from other kinases. Approximately a decade ago, initial *in vitro* studies identified GSK-3β as a negative regulator of the hypertrophic response in cardiomyocytes and examined its role in the processes leading to heart disease. Haq *et al*. demonstrated that the hypertrophic response of cardiomyocytes to hypertrophic agonist stimulation is reduced when GSK-3β with a Ser9 to Ala mutation- a mutation that is not inhibited by Akt-is introduced *via* adenovirus-mediated gene transfer. This study concluded that for cardiomyocytes to exhibit a hypertrophic response, inactivation of GSK-3β is essential. Moreover, specific deletion of GSK-3β in cardiac fibroblasts has been shown to lead to fibrogenesis, left ventricular dysfunction, and excessive scarring in the ischemic heart [104]. Activated glycogen synthase kinase 3 beta (GSK-3β) inhibits heart hypertrophy in response to pressure overload and adrenergic stimulation [101]. Curcumin protects against regional myocardial ischemia/reperfusion injury by activating the RISK pathway and glycogen synthase kinase 3 beta (GSK-3β), while inhibiting p38 mitogen-activated protein kinase (MAPK) and c-Jun N-terminal kinase (JNK), as demonstrated by Jeang *et al*. [105]. To date, no small molecule has been specifically developed to confer cardioprotective effects through the activation of glycogen synthase kinase 3 (GSK).

#### 5.3.4 Interventions Targeting CK1

Members of casein kinase 1 (CK1) play a crucial, conserved role in Wnt/β-catenin signaling, acting as both activators and inhibitors by phosphorylating various pathway components [106, 107]. The anthelminthic drug pyrvinium allosterically activates CK1a, identified as its primary target, although effects on other CK1 isoforms and pygopus have also been noted. Pyrvinium has been shown to block Wnt signaling and provide benefits after ischemia in a mouse myocardial infarction model, with CK1a involvement confirmed *in vitro* [108]. While some studies suggest a STAT3-mediated mechanism, others propose alternative mechanisms affecting PI3K/Akt. Additionally, CK1 isoforms other than CK1a may target different components of signaling cascades or modulate the destruction complex [83, 109, 110]. The inhibitor IC261 can suppress β-catenin transcription and potentially inhibit Wnt signaling in conjunction with specific CK1 inhibitors. Moreover, CK1d/ε may be critical for inducing changes in pluripotent stem cells into cardiomyocytes, with possible inhibition by the p38 MAPK inhibitor SB203580 [84, 85, 111].

#### 5.3.5 Interventions Inhibiting GSK3β

The canonical Wnt signaling pathway, regulated by GSK-3, is crucial for cell signaling. In the absence of Wnt ligands, β-catenin, the key transcriptional effector for Wnt-responsive genes, is phosphorylated by a degradation complex including GSK-3β, leading to its ubiquitination and degradation, which maintains low cytoplasmic levels. Upon Wnt ligand binding to cell surface receptors, the degradation complex is recruited to the membrane, preventing β-catenin phosphorylation. This allows β-catenin to accumulate, enter the nucleus, and activate downstream target genes, indicating that pharmacologic inhibition of GSK-3 can activate the Wnt pathway [104, 112]. Disruption of β-catenin during development and adulthood has implications for cardiac electrophysiology and arrhythmia vulnerability. Wnt/β-catenin specifically regulates genes like Scn5a (encoding the cardiac sodium channel NaV1.5) and Gja1 (encoding connexin 43). β-Catenin also plays a role in cell-cell adhesion at adherens junctions with N-cadherin. In hypertrophic cardiomyopathy, β-catenin accumulates at intercalated discs with concurrent reduction of GSK-3β, indicating that decreased GSK-3 activity may affect electrophysiological reprogramming through β-catenin regulation [113]. Inhibition of GSK-3 with SB216763 has been shown to alter cardiac conduction and create a proarrhythmic substrate in both mouse and human hearts. Research suggests that therapeutic targeting of GSK-3 must consider these potential adverse cardiovascular effects [114, 115].

### 5.4 β-Catenin and Gene Transcription

When β-catenin enters the nucleus, it forms complexes with several TCF/LEF transcription factors and their coactivators, including CBP and p300, leading to the transcription of various target genes such as AXIN2, CD44, and CCND1. This transcriptional regulation is crucial in the development of several diseases. While recent research has focused on designing compounds to target the β-catenin pathway for anti-cancer treatment, there is limited exploration of small molecules aimed at cardiovascular diseases [116, 117]. ICG-001 is a small molecule that specifically disrupts the interaction between β-catenin and the coactivator CREB binding protein (CBP), while leaving the similar interaction with the coactivator p300 unaffected. This selectivity allows ICG-001 to inhibit β-catenin/CBP-mediated transcriptional activation, which is important for stem and progenitor cell differentiation, particularly in the context of neuronal and hematological regeneration. Studies by Sasaki *et al*. revealed that ICG-001 enhances the expression of genes essential for heart regeneration in epicardial cells and modifies Wnt signaling. Lineage tracing experiments confirmed the significance of β-catenin/p300-mediated transcription for the contribution of epicardial progenitor cells to the heart. In trials, female rats treated with ICG-001 for 10 days post-occlusion exhibited a significant improvement of 8.4% in ejection fraction compared to controls [116, 118, 119]. Additionally, research by Xie *et al*. evaluated compounds containing the motif (1,2,4-triazolo [3,4-b]-1,3,4-thiadiazole) for their effects on cardiac regeneration. Their findings showed that Cardiomogen inhibits Tcf/Lef-mediated luciferase activity linked to β-catenin and improves heart recovery in zebrafish with myocardial infarctions, leading to increased cardiomyocyte production and reduced fibrosis [120].

## CONCLUSION AND FUTURE PERSPECTIVE

Targeting the Wnt signaling pathway has seen a notable increase in the number of chemicals during the last ten years. Most of these medicines target this particular branch of the WNT signaling cascade since mutations in various WNT/β -catenin pathway components have been found in several malignancies. However, in cardiovascular disorders, the illness process is more likely to be influenced by changes in the pathway's control than by mutations in its constituent parts. Therefore, farther upstream in the pathway, at the level of WNT proteins or their interaction with the receptor complex, would be the preferred areas of intervention for cardiovascular diseases. For example, in MI models, manipulations in the PORCN and receptor complex have been demonstrated to have a positive impact on wound healing. Additionally, β-catenin-mediated signaling enhanced wound healing, indicating that both β-catenin and non-β -catenin pathways were involved.

The emergence of side effects is a key worry for the worldwide suppression of WNT signaling. Due to their reliance on WNT signaling, a number of rapidly dividing cells, such as those in the skin and intestinal epithelium, could be disrupted by an intervention, which could result in diarrhea or other problems. Remarkably, in cases where these medications were used, such adverse effects have hardly ever been reported. This might be explained by the fact that these epithelial cells only require a certain quantity of WNT signaling, which makes a partial blocking safe for these cells. Naturally, more study on this subject will be required before these substances may be used in patients with success. Future research has an intriguing and promising direction in the context of heart failure and cardiovascular illness, thanks to the investigation of the Wnt signaling pathway. Discovering the functions of the Wnt pathway in the adult heart and developing new therapeutic approaches may provide hope for the millions of people suffering from the debilitating consequences of cardiovascular disease worldwide.

## Figures and Tables

**Table 1:** Some of the key WNT signaling members and cardiovascular disease.

**Wnt Signaling Members**	**Pathway**	**Role**	**References**
Wnt1	β-cathenin pathway	Cardiac neural crest cell development	[55]
Wnt2B	Non-β-cathenin pathway	Inhibition of cardiac progenitor cells	[56]
Wnt3a	β-cathenin pathway	Inhibition of cardiac progenitor cells	[57]
Wnt5a	Non-β-cathenin pathway	Cardiomyocyte differentiation	[58]
Wnt7a	Non-β-cathenin pathway	Conduction system development	[59]
Wnt9b	β-cathenin pathway	Epicardium development	[60]
Wnt11	Non-β-cathenin pathway	Cardiomyocyte differentiation	[61]
Dkk-1	β-cathenin pathway	No correlation with storke severity, inversely associated with cad or atherosclerosis	[62]
Dkk-3	β-cathenin pathway	-	[63]
TCF7L2	β-cathenin pathway	Promote differentiation and inhibit proliferation of SMCs	[64]
LRP5	β-cathenin pathway	Upregulated cytokines and pro-inflammatory genes in HC Lrp^5-/-^mice	[65]
LRP6	β-cathenin pathway	LRP6 highly expressed in VSM, LRP6 mutations impaired LDL clearance	[66]

**Table 2:** Some Wnt inhibitor chemical structure with their targets and effects in cardiovascular disease.

**Compounds**	**Use**	**Target**	**Effect**	**References**
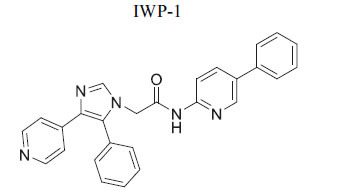	*In vivo* Goltz syndrome	PORCN protein	Inhibition of Wnt3a secretion	[72]
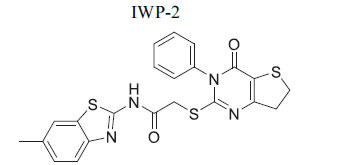	*In vivo* cancer cells	PORCN protein	Inhibition of Wnt1, Wnt2a and Wnt3a secretion	[73]
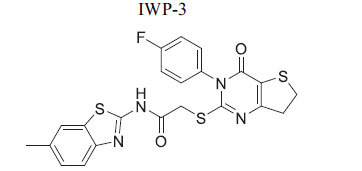	*In vivo* cancer cells	PORCN protein	Inhibition of Wnt1, Wnt2a and Wnt3a secretion	[73]
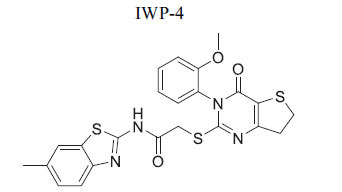	*In vivo* cancer cells	PORCN protein	Inhibition of Wnt1, Wnt2a and Wnt3a secretion	[73]
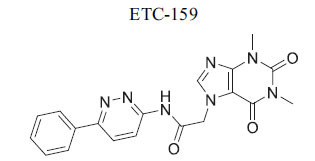	*In vitro* colorectal cancer cells	PORCN protein	Inhibition of Wnt1, -2, -3A, -6, -7B, 28A, -9A, -9B, and -10B secretion	[74]
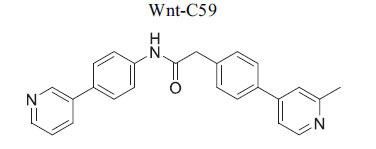	*In vitro* and *in vivo*	PORCN protein	Inhibition of Wnt1, -2, -3A, -6, -7B, 28A, -9A, -9B, and -10B secretion	[67]
OMP-18R5 (mAb)	*In vitro* and *in vivo* (mice	FZD1, FZD2, FZD5, FZD7, FZD8	Inhibition of Wnt-FZD interaction	[75]
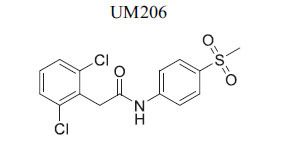	*In vitro* and *in vivo* (mice)	FZD1 and FZD2	Inhibition of Wnt-FZD interaction	[76]
Anti-Sclerostin Ab	*In vitro*	Sclerostin binding to LRP5/6	Prevention of Sclerostinmediated disruption of LRP5/6-FZD complex formation	[77]
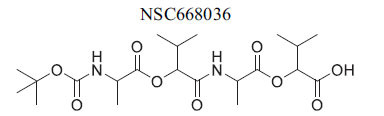	*In silico* and *in vivo* (Xenopys embryos)	DVL PDZ	Blockade of DVL → Inhibition of WNT signaling	[78]
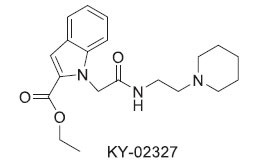	*In silico*, *in vitro*, *ex vivo*, *in vivo* (mice)	DVL-CXXC5	FZD7-DVL PDZ interaction disruption → Inhibition of WNT signaling	[79]
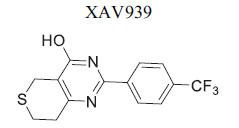	*In vitro* and *in vivo* (mice)	axin	stabilization of axin Enhancement of axin-GSK3b complex formation, Inhibition of Wnt	[80]
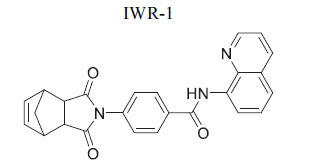	*In vitro* and *in vivo*	axin	Induction of protein levels and stabilization of axin, Enhancement of axin-GSK3b complex formation → Inhibition of Wnt	[81]
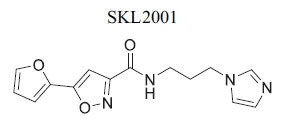	*In vitro*	axin	Disruption of the axin/ β-catenin interaction → Activation of Wnt signaling	[82]
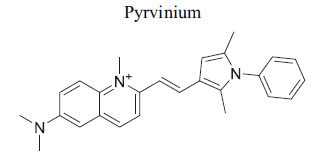	*In vitro* and *in vivo* (Xenopus embryo and mice)	CK1	Induction of CK1a → Enhancement of axinGSK3b complex formation → Inhibition of Wnt Signaling	[83]
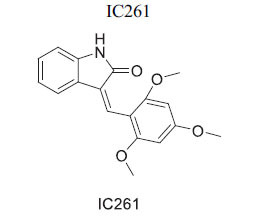	*In vitro*	CK1	Targeting of CK1« → Inhibition of Wnt Signaling	[84]
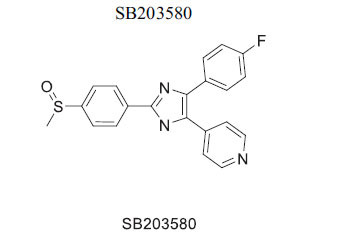	*In vitro*	CK1	Targeting of CK1d/« → Inhibition of Wnt Signaling	[85]
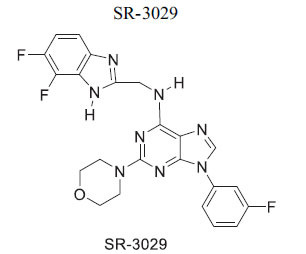	*In vitro* and *in vivo* (mice)	CK1	Targeting of CK1d → Inhibition of Wnt Signaling	[86]
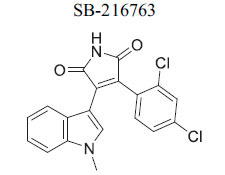	*In vitro*	GSK3β	Inhibition of GSK3β → Activation of Wnt Signaling	[87]
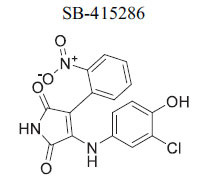	-	-	-	-
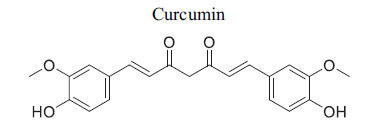	*In vitro*	GSK3β	Akt inhibits p-GSK3β, activation of GSK3β , destruction complex → Inhibition of Wnt Signaling	[88]
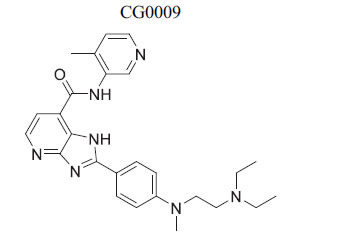	*In vitro*	GSK3β	Induction of Ser9 p-GSK3β and inhibition of Tyr215 p-GSK3β → Inhibition of GSK3β →Activation of Wnt Signaling	[89]
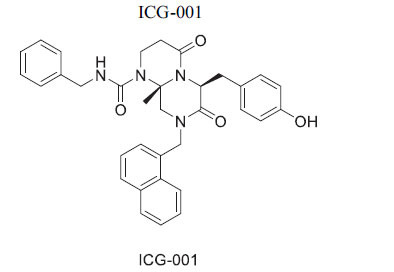	*In vitro*, *in vivo* (mice) and CT	CBP	Disruption of the β-catenin/ CBP interaction → Inhibition of WNT Signaling	[90, 91]

## LIST OF ABBREVIATIONS

APC: Adenomatous Polyposis Coli
BNP: Brain Natriuretic Peptide
CVD: Cardiovascular Disease
DCM: Diabetic Cardiomyopathy
ER: Endoplasmic Reticulum
GSK3: Glycogen Synthase Kinase-3
HF: Heart Failure
IHF: Ischemic Heart Failure
LRP1: Low-Density Lipoprotein Receptor-Related Protein 1
MH: Myocardial Hypertrophy
MI: Myocardial Infarction
SFRP1: Soluble Frizzled-Related Protein 1
TAC: Transverse Aortic Constriction
